# Effects of urine composition on epithelial Na^+^ channel-targeted protease activity

**DOI:** 10.14814/phy2.12611

**Published:** 2015-11-12

**Authors:** Jonathan M Berman, Ryan G Awayda, Mouhamed S Awayda

**Affiliations:** Department of Physiology and Biophysics, State University of New York at BuffaloBuffalo, New York

**Keywords:** Cleavage, ENaC, pH, Protease, Sodium, sodium absorption, Urine

## Abstract

We examined human urinary proteolytic activity toward the Epithelial Sodium Channel (ENaC). We focused on two sites in each of alpha and gamma ENaC that are targets of endogenous and exogenous proteases. We examined the effects of ionic strength, pH and urinary H^+^-buffers, metabolic intermediates, redox molecules, and large urinary proteins. Monoatomic cations caused the largest effect, with sodium inhibiting activity in the 15–515 mEq range. Multivalent cations zinc and copper inhibited urinary proteolytic activity at concentrations below 100 μmol/L. Similar to sodium, urea caused a 30% inhibition in the 0–500 mmol/L range. This was not observed with acetone and ethanol. Modulating urinary redox status modified activity with H_2_O_2_ stimulated and ascorbate inhibited activity. Minimal effects (<10%) were observed with caffeine, glucose, several TCA cycle intermediates, salicylic acid, inorganic phosphate, albumin, creatinine, and Tamm–Horsfall protein. The cumulative activity of ENaC-cleaving proteases was highest at neutral pH, however, alpha and gamma proteases exhibited an inverse dependence with alpha stimulated at acidic and gamma stimulated at alkaline pH. These data indicate that ENaC-targeting urinary proteolytic activity is sensitive to sodium, urea and pH and changes in these components can modify channel cleavage and activation status, and likely downstream sodium absorption unrelated to changes in protein or channel density.

## Introduction

The epithelial sodium channel (ENaC) controls regulated sodium absorption in the mammalian collecting duct (CD). Channel-activating mutations cause hypertension (Schild et al. 1996), and channel activity in vivo and in vitro are established determinants of salt and water balance (McCormick and Bradshaw 2006; Bugaj et al. 2009). Despite its established role, the number of ENaC-activating mutations and their frequency in the general population has been very limited. Besides classical long-acting hormones such as aldosterone which activate the channel, the regulators of baseline channel activity and their variability in the general population are undetermined.

Epithelial sodium channel (ENaC) has been recently shown to be regulated by proteolysis. The largest and most dynamic means of activating the channel occurs by endogenous and exogenous proteolysis at multiple positions in the extracellular loop of the alpha and gamma subunits (Hughey et al. 2004). Despite the presence of some residual activity of the uncleaved channel (Berman et al. 2015), proteolysis and proteolytic enzyme activities have the potential to fully activate and control baseline ENaC activity.

It has been long known that urine is rich in active proteases (Loeper 1922; Farnsworth et al. 1946; MacFarlane and Pilling 1947), and recent proteomics has allowed the identification of many of these proteases (Adachi et al. 2006) and serpin protease inhibitors (Castagna et al. 2011). Over 70 different urinary protease and protease inhibiting molecules have been identified, and some of these such as kallikrein, plasmin, and prostasin have been shown to activate ENaC (Adachi et al. 2001; Passero et al. 2008; Gondzik et al. 2012; Patel et al. 2012). A lot more is known of the general activity and/or function of urinary proteases (Griendling et al. 1993), however, little is known linking changes of urinary protease activity to diseases mediated by changes in ENaC function. Examples of such association exist in Nephrotic Syndrome in which urinary plasmin activity is increased (Candiano et al. 2006). However, the link to ENaC and pathogenesis in vivo is tenuous as elevated plasmin also causes pathogenesis by generating glomerular injury (Baricos and Shah 1991).

A limitation in linking ENaC proteolysis to disease states is the presence of multiple proteases which can act on the channel making analyses of activities of a single protein difficult to decipher. Also, a few biochemical assays exist for measuring protease protein levels and these lack resolution. Existing assays measuring proteolytic activities are also limited in their usefulness as a link to ENaC activation as their substrates are shared by multiple proteases because of the broad protease substrate preference (Gosalia et al. 2005a). The activities of proteases are also measured under controlled and standardized conditions that do not reflect the biological variability in urine. Such differences are important given urinary variability. Variations in components within urine, including examples such as pH, urea, and Na^+^ concentration have the potential to modify activity and to go completely undetected in standardized measurements. Some of these modulators have been examined in purified proteases in nonphysiological solutions (Ru et al. 2000), however, a systematic ENaC-relevant evaluation is lacking.

We systematically examined modulatory changes in urinary proteolytic activity. This analysis was performed with urine collected from human subjects because this represents the entire protease content of the luminal material. We examined effects of pH, salts, chaotropic agents, metabolic byproducts, large proteins, and redox reagents. We focused on activity which is ENaC activation relevant. To do this, we expanded on an amidolytic assay we developed (Awayda et al. 2011) to include ENaC targets in both alpha and gamma subunits. We utilized four peptides with sequences derived from sites endogenously and exogenously cleaved by furin-type proteases (Hughey et al. 2004). We included a fifth ENaC-unrelated substrate, as a control to compare the overall effect on urinary protease activity versus those that can target ENaC leading to channel activation.

We report that urinary ENaC-activating proteolytic activity is markedly inhibited by monatomic cations, with the largest inhibition of ∼80% by sodium and potassium. Urea also inhibited activity by ∼30%. In general, larger effects were observed at RRAR alpha and RKRR gamma substrates. Alpha-cleaving proteases were activated at low pH, whereas gamma-cleaving ones were activated at high pH; with the highest combined activity at pH 7–8. This indicates that acidosis and alkalosis can potentially shift the makeup of the channel to containing mostly active alpha, to mostly active gamma to both. Altogether, these data indicate that changes in urinary composition may underlie an unrecognized mechanism to channel activation and indirectly to enhanced or decreased sodium reabsorption in the CD.

## Materials and Methods

### Urine collection and dialysis

Morning urine (50 mL) was collected from six normotensive individuals, (four male, two female) on no medication. A 20 mL aliquot was set aside and subdivided to allow individual analyses of proteolytic activity, [Na^+^], [K^+^], and osmolarity without multiple freeze thawing. The remaining 30 mL aliquot was combined and immediately dialyzed. This used a 2 kDa slide-a-lyzer cassette (Thermo Sci., Rockford, IL) with a capacity of 30 mL and followed the manufacturer’s instructions. Briefly, 30 mL was inserted into the cassette which was placed for overnight dialysis at 4°C. After a 2× dialysis against 2 L Tris buffer (20 mmol/L Tris, 15 mmol/L Na^+^, pH 7.9, filter sterilized), the dialyzed urine was aliquoted and used for further analysis of proteolytic activity. This procedure was repeated on three separate batches of pooled urine. All collection procedures followed a protocol approved by the University at Buffalo Institutional review board.

### Tamm–Horsfall

Tamm–Horsfall protein was depleted from urine by addition of 1 mol/L NaCl (Tamm and Horsfall 1952) followed by 15 min incubation at −80°C. The precipitate was separated by 15 min centrifugation at 3000 *g*. Precipitate of 0.8 g was recovered per 50 mL of urine, representing 1.6% w/v. NaCl was removed by dialysis as above. Precipitated THP was redissolved into solution in the dialysis buffer.

### Proteolytic activity

Protease activity was measured using an amidolytic assay as previously described (Awayda et al. 2011). Briefly, high-purity peptides (>95% purity) were synthesized coupled at the C-termini to amino-methyl coumarin (Genscript, Piscataway, NJ). 100 *μ*L reactions consisted of 15 mmol/L tris buffer, pH 7.9 (unless otherwise stated), 10 μmol/L peptide, and dialyzed urine or purified protease (5 *μ*L). Fluorescence intensity was measured in a 96-well plate reader (Bio-Tek, Winooski, VT) with excitation emission at 360/460 nm. Reactions were monitored by the appearance of fluorescence after cleaving the peptides from AMC, and were carried out at 37°C. Points were recorded at 10 min intervals and the 2nd to 7th points were used to calculate velocity (slope). Because this calculation eliminates initial fluorescence, any background fluorescence from additional reagents used in these assays did not affect the measurement of reaction rate. However, because the reaction was carried out in 5% urine, reaction rates were slow enough to obtain background measurements for every reaction and in no case affected interpretation of our results.

### Reagents

Kallikrein was purchased from 3H Biomedical (Uppsala, Sweden). All other reagents were of highest research grade purity and were purchased from Fisher Scientific or Sigma-Aldrich. ENaC substrates used were as follows in single letter amino acid code coupled to amino-methyl coumarin (AMC): RRAR-AMC and RSRR-AMC for alpha subunit cleavage at R204 and R178, RKRK-AMC and RKRR-AMC for gamma subunit cleavage at R138 and R181. Other substrates used were PFR-AMC, a kallikrein/low specific trimeric substrate, and AAF-AMC for examining subtilisin activity (Bachem, Torrance, CA). Unless indicated, all reactions were carried out at pH 7.9 Tris buffer containing 15 mmol/L Na^+^.

### Urinalysis

Osmolarity was measured using an Osmette freezing point depression osmometer (Precision Systems, Natick, MA). Urine [Na^+^] and [K^+^] were measured using an Instrumentation Laboratory (Bedford, MA) flame photometer. pH was measured with Denver instruments meter (Denver CO) using a three-point (pH 4–10) calibration.

### Statistics

Significance was determined at *P* < 0.05, Student’s *t*-test.

## Results

### Amidolytic assay

**Figure 1:**
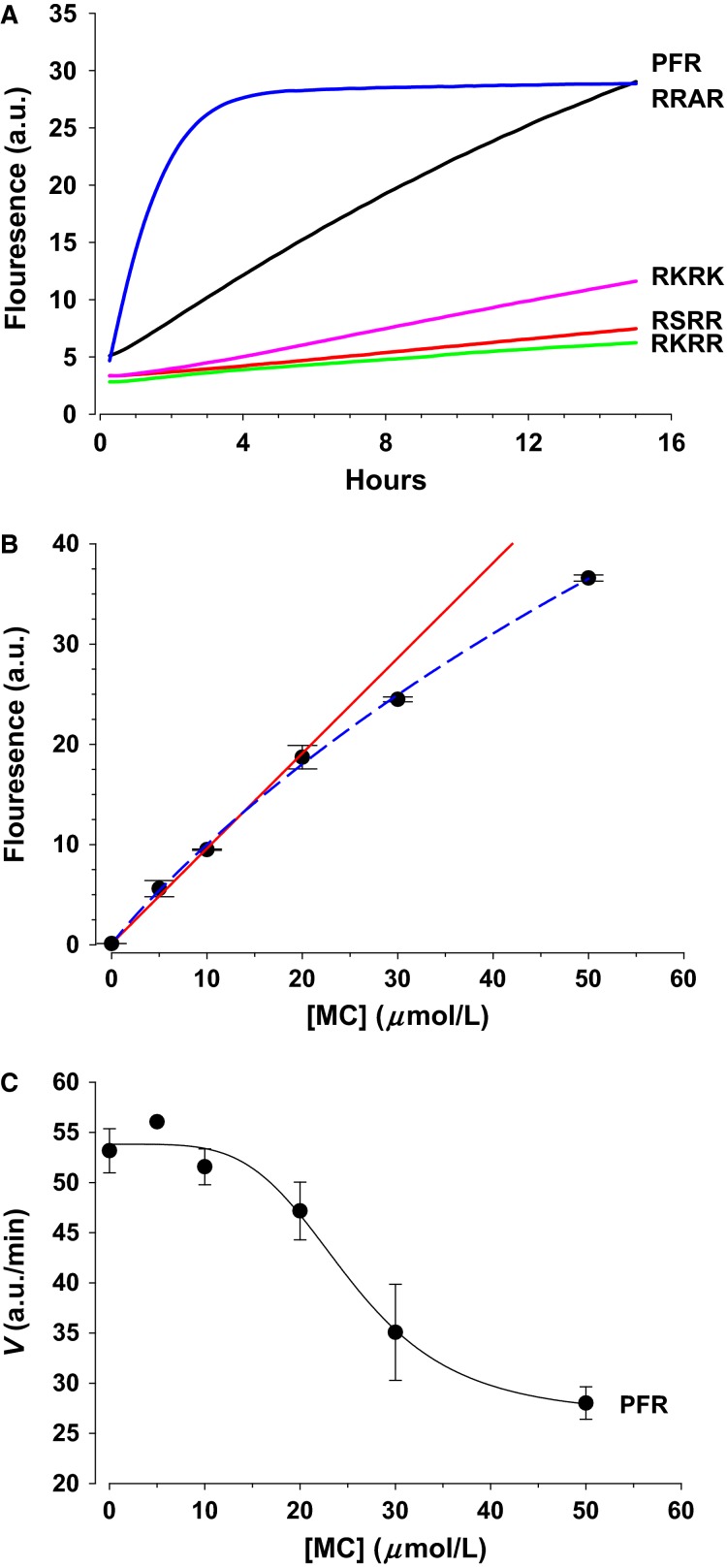
Urinary protease ENaC directed amidolytic assay and optimization. (A) Example progression curves for 4 ENaC peptides and PFR, points taken from experimental data in 10 min intervals. Peptides were used at 10 μmol/L substrate and urine was used at 5% of final volume. The maximal slope of the reaction was calculated from the first 3–5 points and used as an index of rate in arbitrary units/time. (B) Coumarin fluorescence as a function of concentration. Fluorescence deviates from linearity above 20 μmol/L. (C) Reaction velocity of PFR hydrolysis in units/time and the effect of exogenous methyl coumarin. Inhibition was observed as a significant attenuation of rate at concentrations greater than 15 μmol/L. *N* = 4–6.

We have previously described a 4 a.a. ENaC specific amidolytic assay (Awayda et al. 2011). We expand on this assay to test more ENaC ligands and to examine the effects of urine composition on urinary protease activity at two sites for each of alpha and gamma ENaC. We used four amino acid substrates to mimic those occurring in ENaC in vivo as the minimal but specific cleavage sequence (Awayda et al. 2011). Figure1A shows example reaction progression curves generated by cleavage of substrates by 5% human urine. We have used similar urine/reaction volumes (Awayda et al. 2011) and this avoids the nonlinearity which occurs by substrate depletion at high enzyme concentrations, for example, 100% urine. Reaction velocities were calculated from the initial slopes as previously described (Awayda et al. 2011). Highest activities were observed for the less specific trimeric PFR, followed by RRAR for alpha and RKRR for gamma ENaC.

We optimized the assay using two criteria. We examined the effect of self-quenching of the reaction product – methyl coumarin (MC). As shown in Figure1B quenching is observed as a deviation from linearity at concentrations above 20 μmol/L. We also examined if MC can modify the reaction rate. This is because warfarin which is a structurally related molecule affects clotting which is proteolytic activity dependent (Li et al. 2004). As shown in Figure1C, MC at concentrations greater 20 μmol/L inhibited the reaction velocity for urinary proteolytic enzyme hydrolysis of the peptide PFR. For these reasons, we selected a peptide concentration of 10 μmol/L. This assures that at no point of the reaction – even up to full completion – is the MC concentration capable of affecting reaction fluorescence.

**Figure 2:**
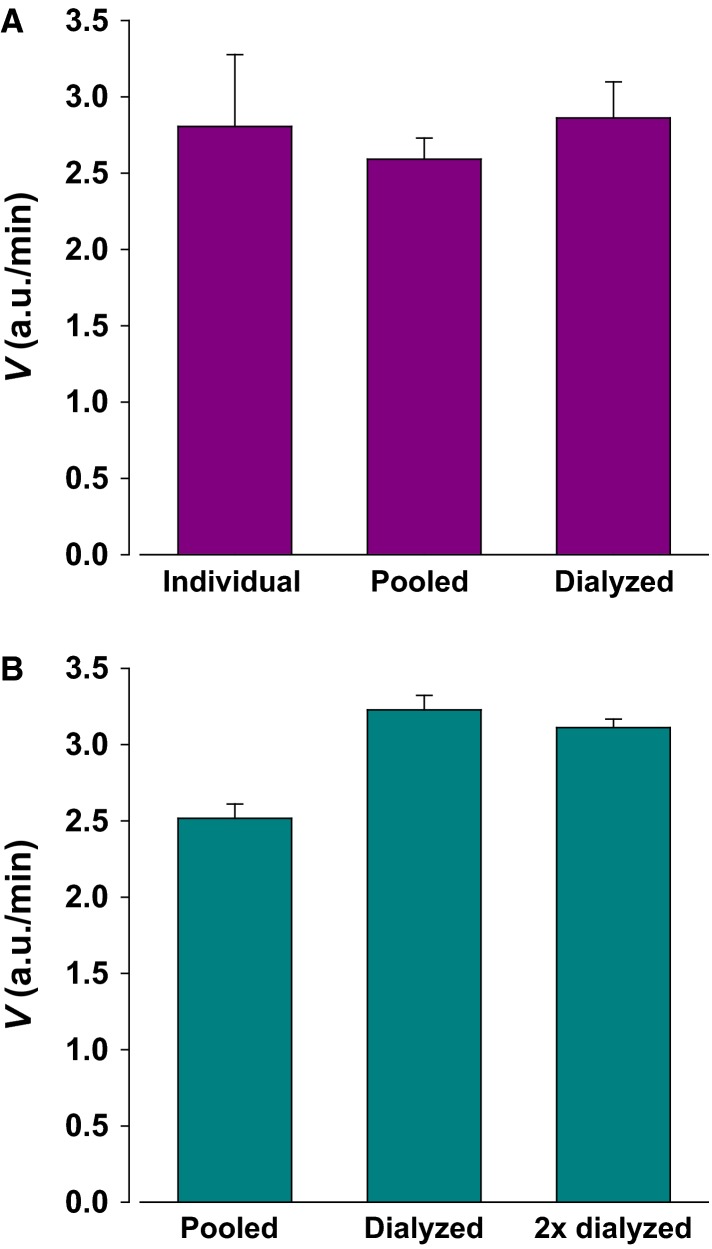
Urine pooling and dialysis do not inhibit activity. Proteolytic activity of human urine for PFR was measured to determine the effect of urine pooling and potential loses during dialysis. (A) Mean activity of six individual urine samples was the same as the average measured activity of pooled urine showing that dialyzed urine did not lose activity. (B) Paired dialysis indicated that dialyzed urine exhibited an increase of activity despite the potential small losses by binding of enzymes to dialysis membrane. This indicates the dialysis of small molecules which cause inhibition of activity. A second dialysis did not further affect activity indicating minimal protein loss by binding to membrane and minimal degradation during the dialysis procedure. Data from 3 repeats.

In ongoing experiments, we examined variability of urinary proteolytic activity between subjects and within the same subject in response to diet, salt, and water balance as well as diurnal changes. To eliminate variability and to establish a baseline, we collected morning urine from six healthy normotensive subjects and pooled it for further analysis. We standardized pooled urine by dialysis in 15 mmol/L Tris buffer. Urinary Na^+^ ranged from 30.4 mEq to 194.5 mEq and averaged 94 mEq. Urinary K^+^ ranged from 28.5 mEq to 66.1 mEq and averaged 47.3 mEq. After dialysis Na^+^ was 15 mEq and K^+^ was 0 mEq. Dialysis reduced pooled urine osmolarity from 524 mOsm to 80 mOsm. Figure2A shows that mean activity of individual urine is not different from that of pooled urine. It also shows that both activities match that of urine which was pooled then dialyzed.

We then examined the recovery of activity after dialysis. During dialysis two sources can result in potential losses of activity. First, enzyme binding to dialysis tubing, and second, partial degradation during the overnight dialysis process at 4°C. As shown in Figure2B, in paired experiments the activity of dialyzed urine is actually higher than that of pooled urine and is nearly unchanged in twice dialyzed urine.

### Salts

The final composition of urinary electrolytes is variable and these changes are physiological and pathological indicators. Moreover, the concentration in various water regulatory segments of the nephron (such as CCD) varies in hypo or hyperosmolar conditions. The activity of many enzymes is known to depend on small molecule cofactors. We have previously published data showing enhanced proteolysis of ENaC under low Na^+^ conditions in the *Xenopus* oocyte system (Berman et al. 2015). For these reasons we examined the effects of common urinary salts. We tested the effects of Na^+^, K^+^, Ca^++^, Cu^++^, and Zn^++^ salts to represent both mono and divalent ions. The concentrations tested included their normal physiological range, with some values outside this range.

**Figure 3:**
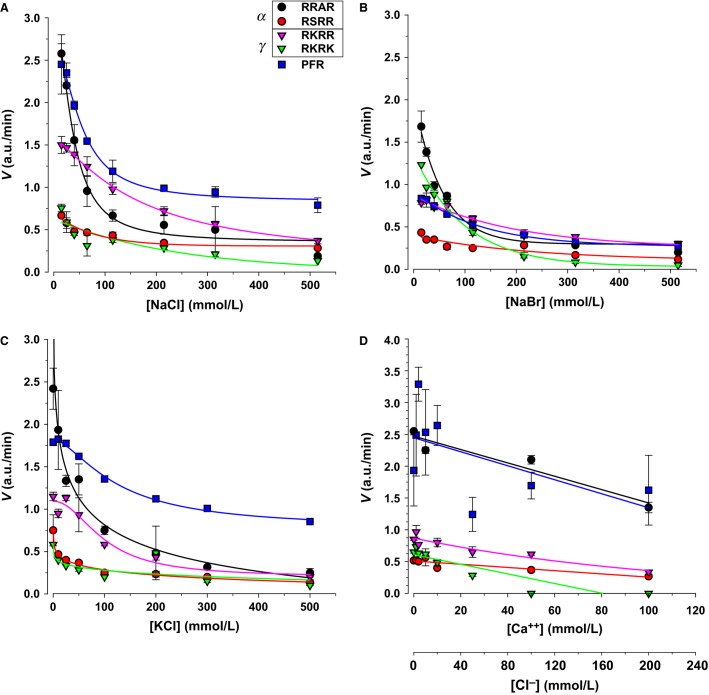
Monovalent cations markedly inhibit urinary protease activity. (A) Increases of NaCl by 0–500 mmol/L (above the baseline of 15 mmol/L from the assay buffer) caused progressive inhibition of the activity of all substrates. Inhibition of ENaC substrates was largest in RRAR and RKRR. PFR was also inhibited, although it exhibited a different response than any of the other ENaC substrates. (B and C) Inhibition of ENaC substrates by NaBr and KCl was similar to that with NaCl and indicate that the major inhibitory role is observed with the cation. (D) CaCl_2_ at physiological millimolar levels did not affect proteolytic activity. At much higher concentrations some effects were observed which were relegated to inhibition of RKRR and RKRK, the two gamma cleavage sites. Even at high levels, the effects of CaCl_2_ were minimal indicating that the inhibition observed with NaCl and KCl were likely mediated by the cations. *N* = 4–6. In this and all following figures, standard error is smaller than point size in data with no visible error bars, and PFR activity was scaled to allow plotting with the ENaC substrates.

Sodium varies in a large range based on the levels of natriuretic and diuretic hormones from as low as 40 mEq/L to nearly 300 mEq/L (Berne et al. 2008). The effects of this salt are shown in Figure3A and B. We compared the effects of chloride and bromide sodium salts. These were similar indicating that the major effect is likely due to the cation. This is consistent with results below. Increasing [Na] inhibited activity for all substrates. The largest effect was on RRAR the 2nd cleavage site in alpha ENaC. This >90% inhibition has the potential to completely abrogate cleavage of this subunit with increasing sodium concentration. Inhibition of RKRR the 1st cleavage site in gamma was 75%, also potentially abrogating this subunit’s cleavage. The overall effect is that as the [Na] increases, activity toward cleaving ENaC – in normal healthy individuals – is decreased leading to reduced absorption and increased Na^+^ excretion.

Similar responses were seen with K^+^ (Fig.3C) although the changes were not as large as Na^+^. Based on current Western diets, K^+^ excretion rates are ½ to 1/3rd those of Na^+^ (Berne et al. 2008). This indicates that excess intake of either salt can lead to diuresis and natriuresis. This may cause a sodium wasting disadvantage in high potassium diets that must be compensated by other mechanisms.

Divalent ions also vary appreciably in urine. Calcium is in the normal physiological range of <15 mEq/L (Berne et al. 2008). At these levels there were no consistent effects on any of the substrates (Fig.3D). However, at supraphysiological concentrations of this salt, an inhibition of PFR, RKRK, and RKRR were observed. This is likely an effect of the high [Ca^++^] as the data with bromide and chloride sodium salts provide strong evidence against a role of the anion. These results also indicate that ENaC-acting human urinary proteases are very different than trypsin type S1 proteases which are known to be sensitive to calcium (Sipos and Merkel 1970).

**Figure 4:**
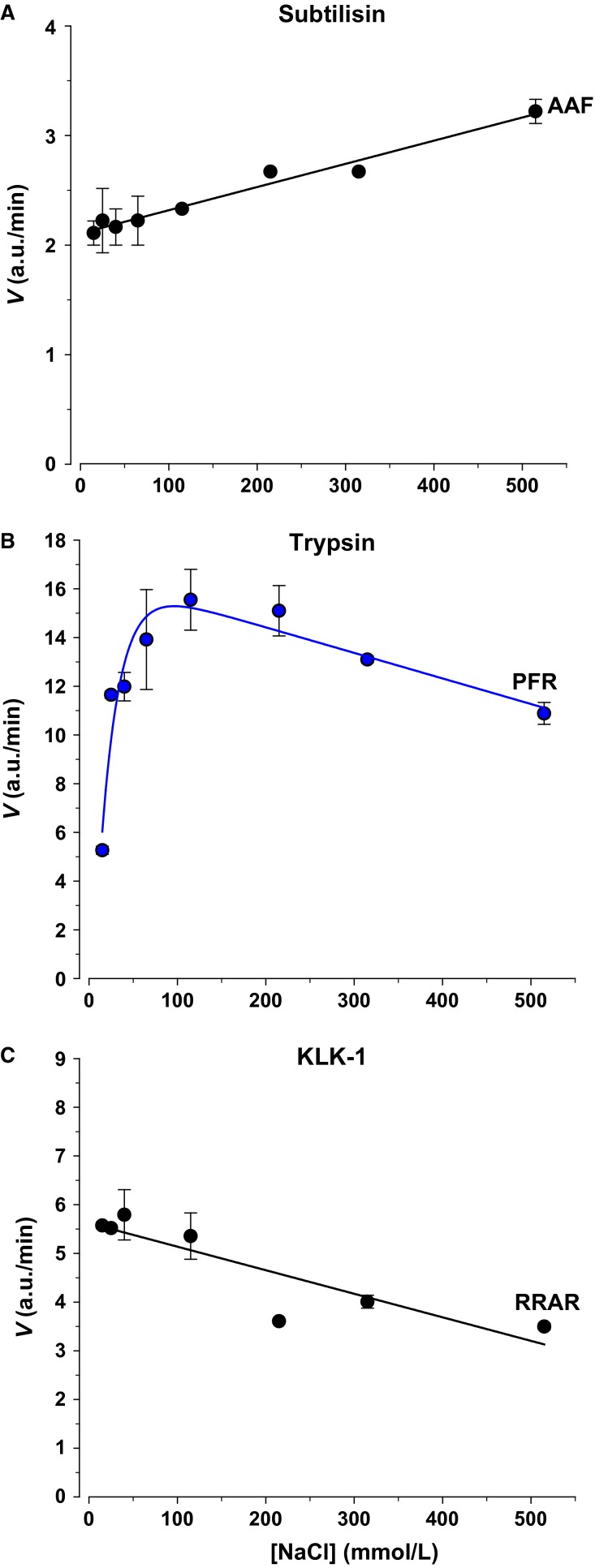
Effect of NaCl on proteolytic activity of purified enzymes. (A) Activity of the S8 serine protease Subtilisin Carlsberg was stimulated by increasing [Na]. (B) Activity of the S1 protease Trypsin was also stimulated by increasing [Na]. This stimulation was biphasic and much more marked than in (A). Both enzymes were assayed at 0.5 *μ*g/mL and their respective substrates were AAF and PFR. (C) Kallikrein1 (0.1 *μ*g/mL) cleavage of RRAR was inhibited by NaCl.

The responses observed above are the average effect observed for pooled normal individuals. It is expected that individual variability in protease composition may lead to a different response to sodium, and possibly a stimulation as observed for some purified proteases (Fig.4). Such differences may end up being important in the response to urinary electrolytes in persons with renal disease especially given the propensity of changes of urinary protease levels to accompany such diseases (Baba et al. 1986).

**Figure 5:**
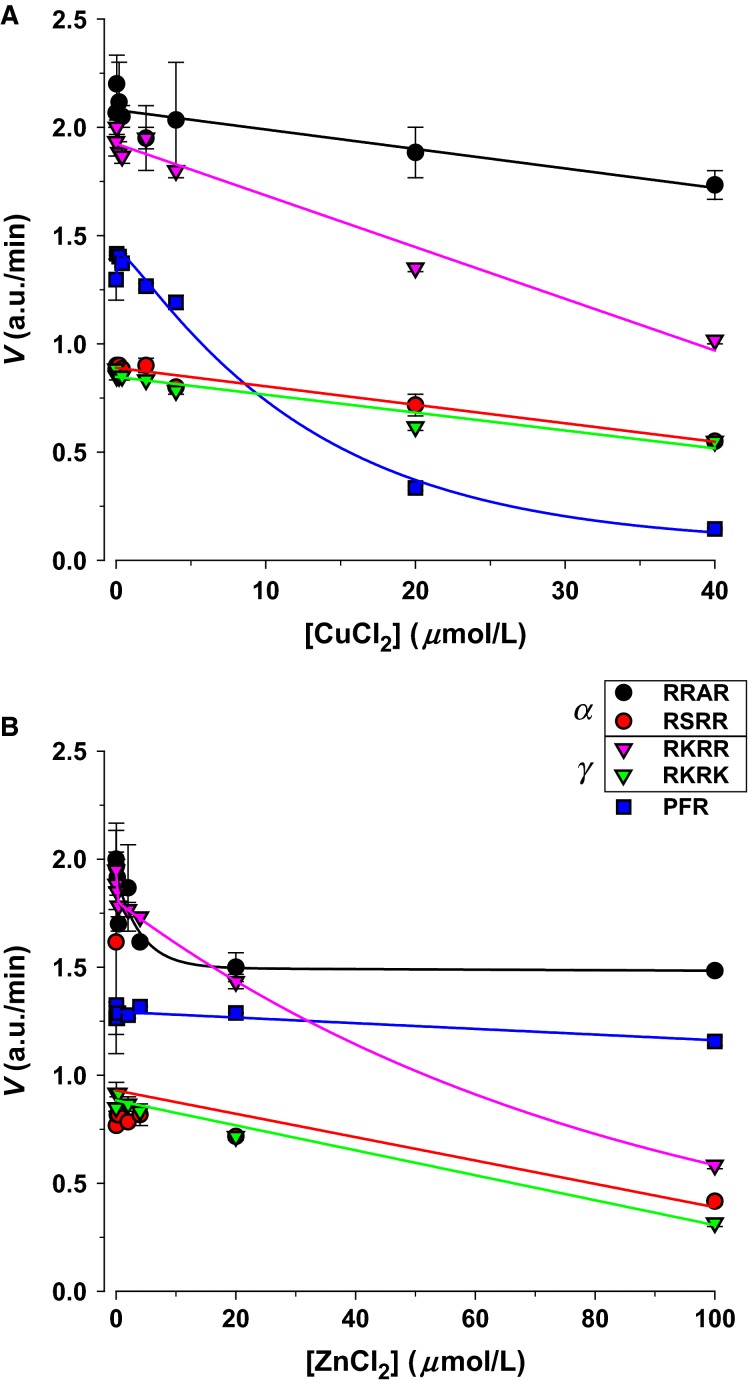
Transition metals inhibit urinary protease activity. (A) Significant inhibition of all proteolytic activity was observed with CuCl_2_ in the range from 0 to 40 μmol/L. The largest inhibition of ENaC substrates was also observed with RRAR and RKRR, similar to that with monovalent cations above. (B) Significant inhibition was also observed with ZnCl_2_ in the range from 0 to 100 μmol/L. The response of ENaC substrates was similar to those with copper, although the effect on PFR was completely absent. These indicate effects of transition metals on ENaC substrates which are likely unrelated to the effect of monovalent cations.

Given the minimal effect of chloride we examined the effects of additional urinary cations. Zinc and copper are trace metals with significance to homeostasis and enzymatic activity (McCall et al. 2000). These ions are known to affect some cellular proteases. In their micromolar urinary levels (copper 20–50 *μ*g/day [Brewer 2002], zinc 300–600 *μ*g/day [McKenzie 1979]), both metals ions caused appreciable inhibition of proteolytic activity (Fig.5). Both caused the largest inhibition in gamma ENaC-acting proteolytic activity. This 40–70% inhibition can markedly affect the cleavage status of this subunit leading to changes in channel activity. These changes were specific to ENaC-acting proteases and unlike those of PFR proteases.

### Denaturants

**Figure 6:**
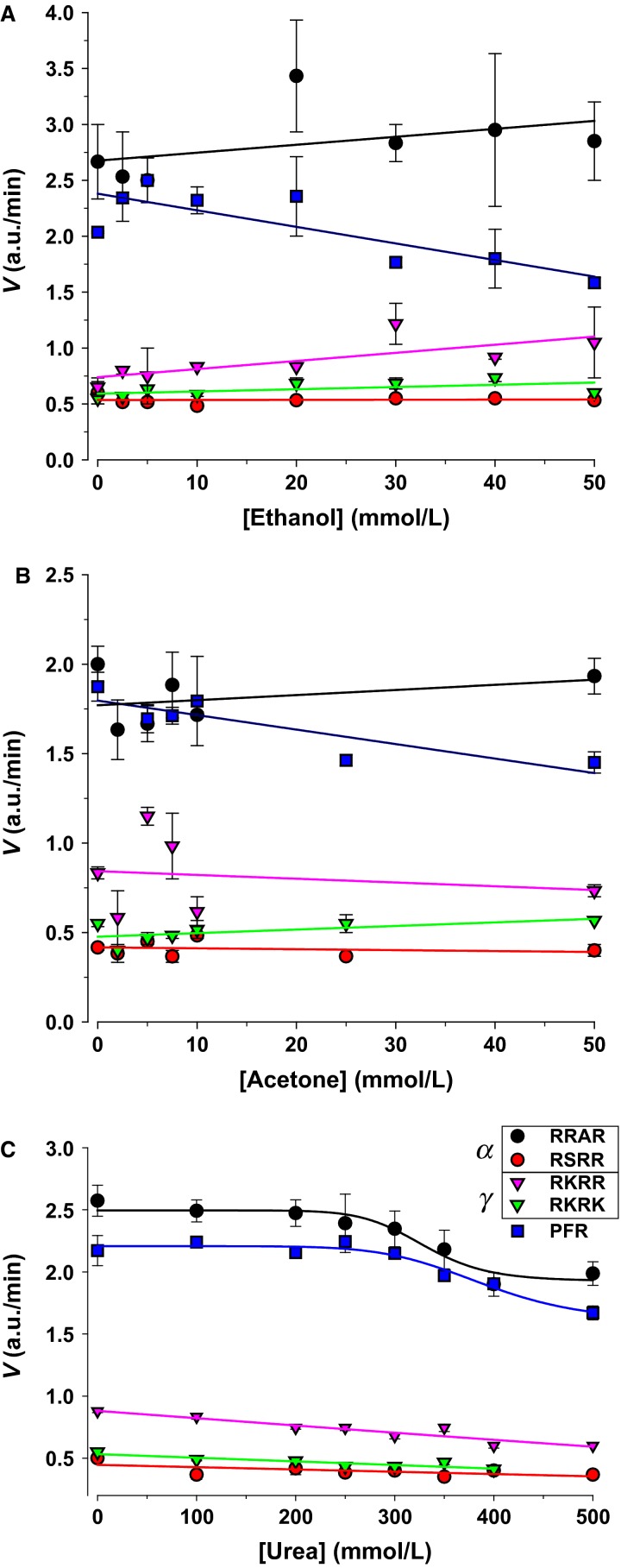
Effects of chaotropic nonionic denaturants on urinary proteolytic activity. The solvents and metabolic intermediates ethanol and acetone, as well as urea were used to test their effects on urinary protease activity. (A and B) Ethanol and acetone in the range 0–50 mmol/L had minimal effects on activity. (C) Urea weakly inhibited cleavage with appreciable effects on RRAR ENaC substrate at concentrations >300 mmol/L.

A possible effect of salts is their ability to change water molecules order (Hofmeister 1888; Melander and Horváth 1977), in addition to acting as a binding partner to some enzymes. To further test this we examined the effects of ethanol, acetone, and urea. Urinary ethanol can reach 20 mmol/L (Tsukamoto et al. 1989). Acetone is found in urine and represents the simplest ketone, which can markedly increase in ketoacidosis (Owen et al. 1982). Neither ethanol nor acetone (Fig.6A, B) produced a significant inhibition of ENaC-acting proteases.

Urea is a known protein denaturant and can be found in urine up to 500 mmol/L. Urea inhibited RRAR proteolysis, but had minimal effects on RSRR, RKRR, and RKRK (Fig.6C). This indicates that the mechanism of inhibition by cations is specific and not shared by other nonionic chaotropic agents (those which change water order).

**Figure 7:**
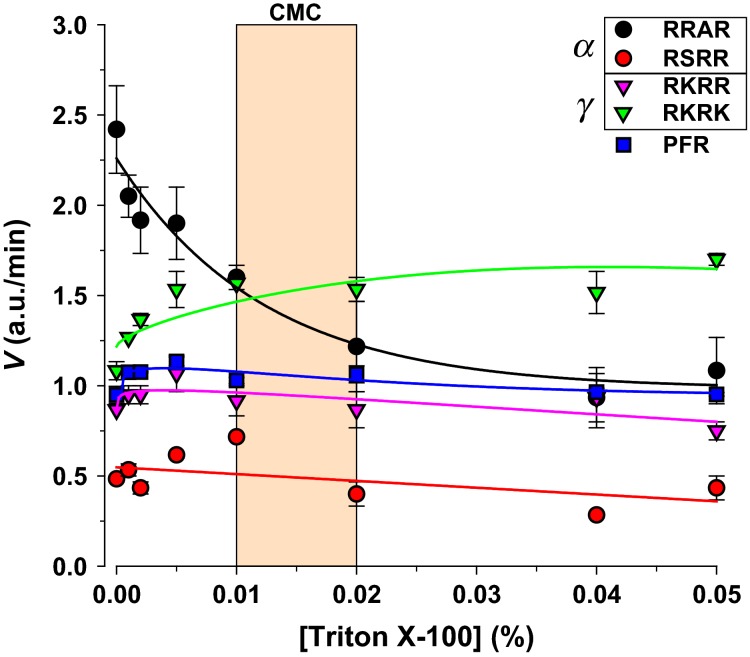
Effects of the nonionic detergent Triton X-100 on Urinary Protease Activity. Triton exhibited paradoxical stimulation of RKRK and inhibition of RRAR ENaC substrates. Most of these effects were observed at the critical micellar concentration of 0.01–0.02% indicating a possible effect on lipids associated with urinary proteases. All other substrates were unaffected.

Another means of modify protein structure involves interactions with urinary lipids or micelles. To test this we examined the effects of the nonionic detergent Triton X-100. Weak detergents have long been used as enhancers for some enzymes (Dennis 1973), and have been shown to increase amiloride sensitive current in isolated toad bladder (Li et al. 1986). As shown in Figure7 Triton X-100 at levels near the critical micellar concentration caused 50% inhibition of RRAR, 50% stimulation of RKRK and no effect on other ENaC-acting proteases. These results may indicate a role for lipids as inverse modulators of RRAR and RKRK-acting proteases. More importantly, these results also indicate the possibility of proteolytic stimulation by small molecule modulators.

### pH

The activity of many enzymes is highly pH dependent. Proteases are also classified based on their optimal pH into acidic, neutral or alkaline proteases. Normal urinary pH is in the range 5–7 and is highly diet dependent (Remer and Manz 1995; Welch et al. 2008). Periods of exercise can also cause production and excretion of excess acid equivalents. The kidneys deal with this by utilizing nonvolatile ammonium and phosphate buffers in addition to secretion as free H^+^.

**Figure 8:**
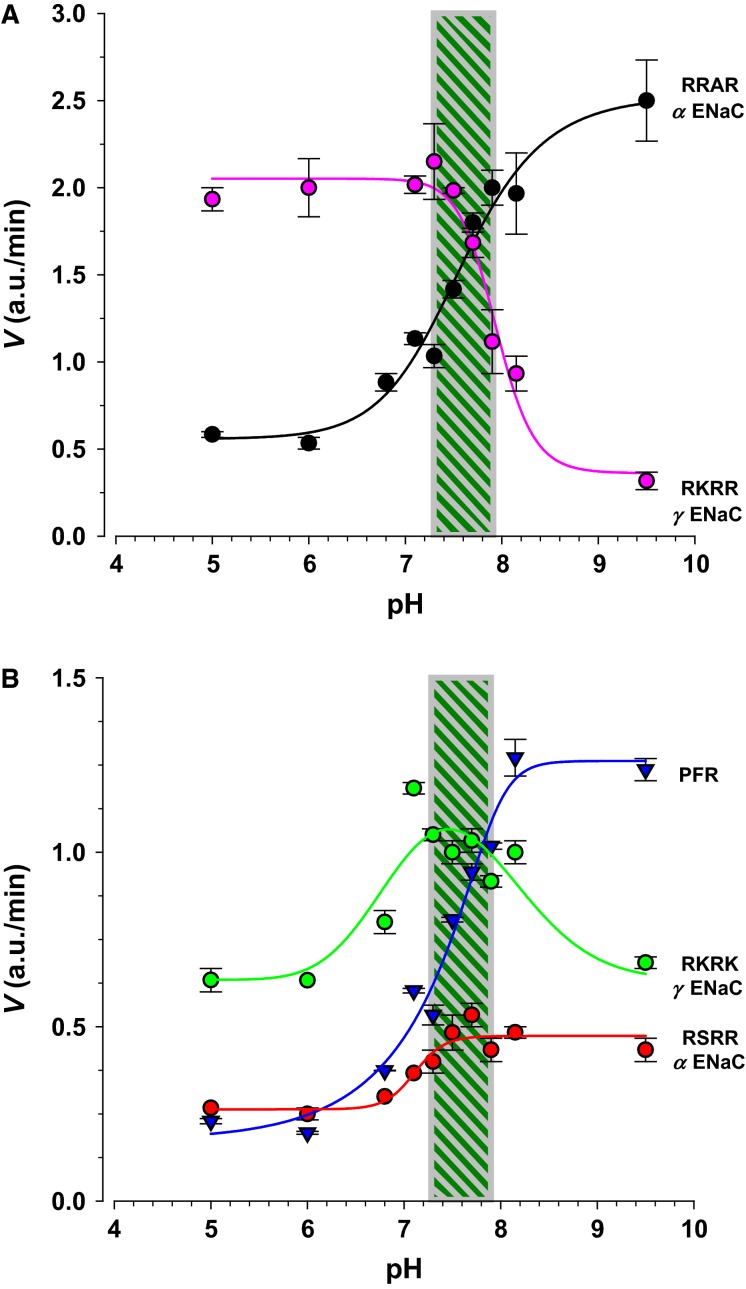
Effects of pH on urinary protease activity. ENaC substrates exhibited opposite effects of pH and the data were subdivided into two groups based on the similarity of the response (inhibition or stimulation) throughout the pH range. (A) The substrates RRAR and RKRR, which were also the most sensitive to monovalent cations, exhibited nearly mirror image responses throughout the pH range. RRAR and RKRR were stimulated by alkaline and acidic pH, respectively. An IC_50_ was calculated to represent the midway point through this relationship. The shaded area represents the pH between the two IC_50_s and the region of high activity for both substrates. (B) The ENaC substrates RSRR and RKRK also exhibited inverse relationship to pH, resulting in a similar peak of activity between pH 7.3 and 7.9. The effect of pH on coumarin fluorescence can be ignored since the data summarize reaction slope or 1st derivatives which would be unaffected by pH induced shifts to the entire progression curve.

The effects of pH are shown in Figure8. RRAR and RKRR which represent alpha and gamma ENaC exhibited opposite responses to acidic and basic pH. The IC_50_ for RRAR was pH 7.3, and that for RKRR was 7.9 (Fig.8A), defining a range of maximal combined activity centered at 7.6. This indicates that full channel activation and cleavage of both subunits is only observed during a narrow pH range and at both extremes more than a fivefold change in cleavage of each subunit can be encountered.

A similar pH window was also observed for the other ENaC substrates RSRR and RKRK (Fig.8B). These changes were smaller and in the range of twofold. The shape of the response was different indicating different complement of proteases acting on these substrates. These data present an interesting area to examine in disease states where the distribution of ENaC-acting proteases might change leading to a differential response to pH and out of optimal activation or inhibition.

**Figure 9:**
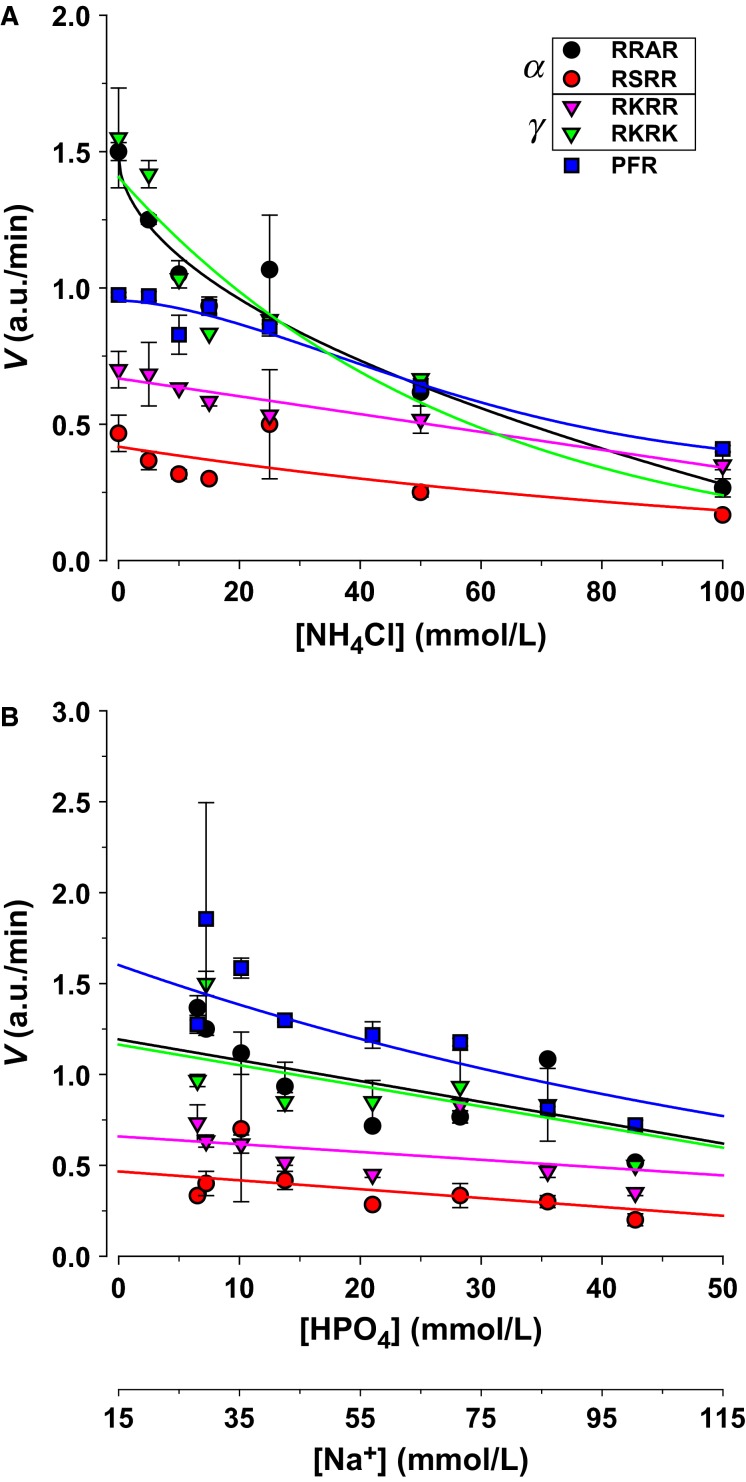
The urinary pH buffers ammonium and phosphate inhibit ENaC targeted proteolytic activity. (A) Ammonium caused inhibition of all ENaC targeted activity with marked attenuation to nearly 80% inhibition of RRAR and RKRK activity by 100 mmol/L ammonium. (B) Inhibition by phosphate was less marked. Given the changes in Na with Na_2_HPO_4_, it is likely that these changes were elicited by the inhibitory effects of this cation. Both effects occurred with minimal changes to pH as measured, and as evident from the differences from the responses observed in Figure8.

The effects of the two main urinary buffers are shown in Figure9. Ammonium caused inhibition of all proteolytic activity with the largest effects of 80% decrease for RRAR and RKRK (Fig.9A). This indicates that acidosis would cause marked effects to sodium transport via a two-fold effect on ENaC cleavage: an effect of free protons, and an effect of ammonium ions, the main urinary pH buffer. This also further highlights the importance of cations in disrupting proteolytic activity.

Similar to ammonium, inorganic phosphate represents an important urinary pH buffer. Although the normal range is ∼20–40 mEq/L (Berne et al. 2008), phosphate concentrations can increase multiple folds in cases of metabolic acidosis. A small inhibition was observed with increasing phosphate concentration (Fig.9B). Given the linearity and similarity of the response to that observed in Figure3, this likely represents the response to changing the Na^+^ concentration in these experiments.

### Tamm–Horsfall protein

**Figure 10:**
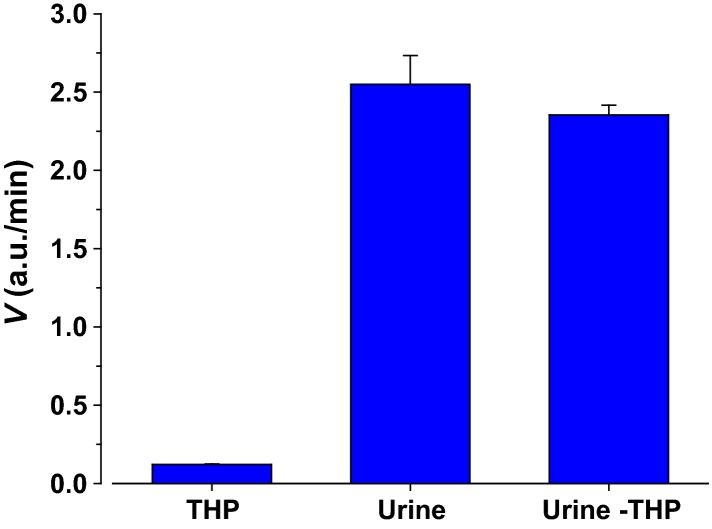
Lack of effects of Tamm–Horsfall protein on urinary protease activity. THP was depleted from urine, and the effect on urinary proteolysis measured. Fraction marked THP indicates that the precipitated protein was suspended in assay buffer at the same concentration as that originally found in this sample of urine. This fraction exhibited little residual activity as compared to fraction marked urine which was untreated control sample. Urine-THP was the same urine pool but one which had THP depleted as described in Methods. This fraction also exhibited similar activity to urine indicating that the presence or absence of THP does not affect activity. Peptide assayed was PFR to maximize changes.

Urine contains many secreted proteins. Tamm–Horsfall protein (THP)(Tamm and Horsfall 1950) is the most abundant protein in urine and its function is not fully elucidated (Devuyst et al. 2005; Padmanabhan et al. 2014). We tested the effects of THP on urinary protease activity. Urine was depleted of THP as described in Methods. The control sample was treated in the same manner except THP was not separated by centrifugation and allowed to redissolve into solution following dialysis. As shown in Figure10, there was no difference in activity between dialyzed urine with and without THP. The THP precipitate was also resuspended into solution and it exhibited minimal activity indicating no interaction between THP and ENaC-acting proteases and also no entrapping of proteases by the polymerized THP.

The effect of albumin and creatinine was also tested. They represent other large urinary constituents and both have the capacity to bind other substrates. Albumin has also been used to increase the rate of many restriction enzymes (Williams et al. 1996) possibly by molecular crowding. These were without effect on proteolytic activity (not shown).

### Additional experiments

#### Metabolic intermediates

**Figure 11:**
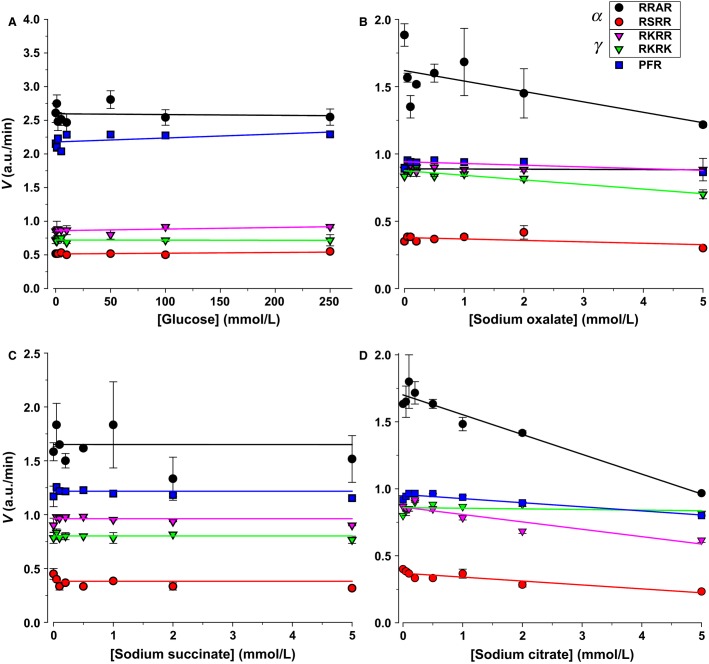
Effect of metabolic intermediates on urinary protease activity. (A) Glucose had no effect on urinary proteolytic activity. (B and C) The di-carboxylates sodium oxalate and sodium succinate also had no significant effect on proteolytic activity. (C) The tri-carboxylate sodium citrate appreciably inhibited RRAR and RKRR by 70% and 40%, respectively. These effects were unrelated to sodium given the low concentration of this cation.

The effects of several TCA cycle and metabolic intermediates were tested. Glucose can be found in diabetic urine and is also a reducing agent potentially capable of reacting with disulfides in urinary proteases. Oxalic, succinic, and citric acids represent di- and tricarboxylic acids and TCA cycle intermediates. These agents did not have significant effects on activity (Fig.11), with the exception of a 70% and 40% decrease of RRAR and RKRR with citrate. These are unlikely a sodium effect given the low millimolar concentration, and indicate potential effects of some tricarboxylate anions similar to that observed with ammonium.

### Caffeine, salicylate, ascorbate, H_2_O_2_

**Figure 12:**
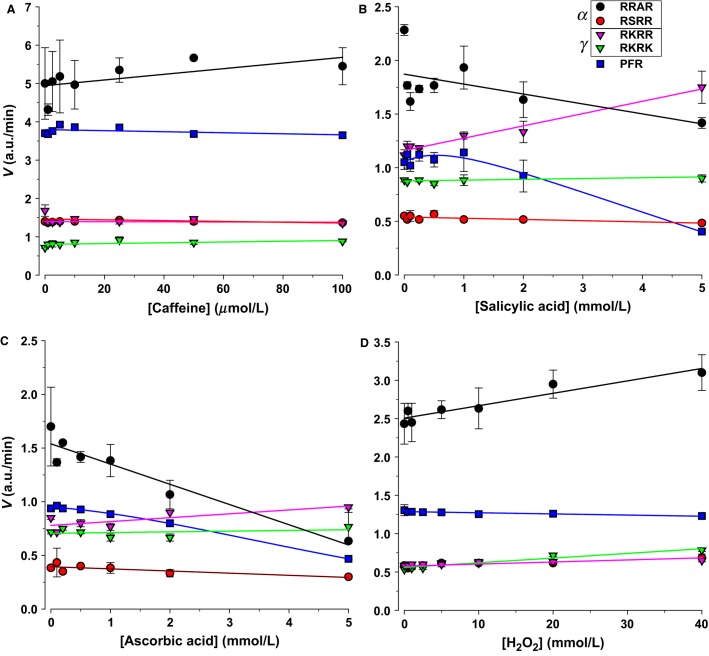
Effect of drug metabolites on urinary proteolytic activity. (A) Caffeine had no significant effect on proteolytic activity. (B) Salicylic acid caused stimulation of RKRR and inhibition of RRAR. The increase of RKRR of 55% was not observed with other maneuvers and specific to salicylate. (C) The antioxidant ascorbic acid inhibited RRAR by >60% and had little effects on all other ENaC substrates. (D) The oxidizing agent H_2_O_2_ exhibited opposite effects to ascorbate on RRAR leading to an ∼25% stimulation and no appreciable effects on the other remaining substrates.

Many commonly ingested drugs or their metabolites are excreted in urine, and some of these are reactive species that can affect oxidation reduction reactions. We selected caffeine, salicylate, ascorbate, and hydrogen peroxide. Their effects are shown in Figure12. Caffeine has been measured up to 60 μmol/L in human urine (Birkett and Miners 1991), and had no significant effect. Salicylate, a common drug, is excreted in urine with a half-life of one hour (Macpherson et al. 1955). It caused a small decrease of RRAR and a 55% stimulation of RKRR. The antioxidant ascorbate is also found in urine and it caused a >60% inhibition of RRAR proteolysis. This was opposite to the 25% stimulation found with the oxidizing agent hydrogen peroxide. Altogether, these indicate potential effects of redox agents and commonly ingested molecules or their byproducts on activity. They also implicate a feedback role of redox reagents on CD Na^+^ transport given the downstream change in these signaling molecules in response to changes in renal hemodynamics and flow.

## Discussion

### Activation of ENaC

Alpha and gamma ENaC are cleaved in their extracellular loops (Hughey et al. 2004). Some cleavage occurs intracellularly, however, uncleaved subunit protein can reach the plasma membrane where it is sensitive to luminal urinary proteases. A correlation has been observed with increased activity of a limited number of proteases, for example, plasmin, and enhanced ENaC activity and sodium retention (Svenningsen et al. 2012). This occurs because cleavage modifies the channel from nearly inactive to nearly fully active. Thus, assessment of urinary proteolytic activity and more specifically ENaC specific activity can provide an index of changes in ENaC function and CD sodium absorption. We report the presence of proteases for all four sites and therefore the capability to cause complete channel activation from the extracellular urinary space. We also report large changes in proteolytic activity based on modulation by components in urine.

### Urinary proteases

The number of urinary proteases and their broad substrate specificity preclude an assessment of the activity of a single urinary protease. Considering that proteases can act on self or other proteases to activate or inhibit, the combinations stemming from having 40+ enzyme and enzyme inhibitors make it impossible to correlate mild changes in activity of a single protease to an end effect such as sodium balance. As we demonstrate, a better option is a reverse approach where cumulative activity to an end substrate or sequence is examined. This option is superior providing a specific substrate is selected; in this case one which is ENaC relevant.

### Protease specificity

A wealth of research on the activity of purified proteases has indicated an overall sequence preference for cleavage. Some proteases, for example, trypsin, exhibit a single amino acid recognition site, whereas others, for example, furin, exhibit a 4 amino acid recognition site. These data are also corroborated with peptide library experiments (Gosalia et al. 2005b) indicating that most protease activity can be more specifically described by 4 amino acid peptides. This is likely the result of the recognition motifs of these proteases being 4 a.a. or less. In this case, a substrate longer than 4 a.a. is not necessary to achieve specific results, and longer substrates can actually interfere with the assay (M. S. Awayda, unpubl. data). The longer peptides may also have additional 3D structure not present in a full protein resulting in diminished binding affinity. In this case, the ideal substrate for this assay is the shortest possible peptide that is specific for the known cleavage motifs in ENaC.

This is illustrated in our experiments by examining the 3 and 4 amino acid substrates. In this case, PFR has been described to be cleaved by many enzymes including tissue kallikrein (Bourgeois et al. 1997), various other kallikreins (1, 2, 5, 8, and 12) (LeBeau and Craik 2012), soybean trypsin like enzyme (Nishikata 1985), and human multicatalytic protease- MCP- (Ustrell et al. 1995). In our hands it also exhibits cleavage by trypsin and subtilisin (M. S. Awayda, unpubl. data). More importantly, and irrespective of the enzymes targeting PFR, this substrate is not ENaC specific. In this case the high activity of PFR, which is >10-fold higher than any other 4 amino acid substrate examined, indicates that it likely represents an integrated index of many urinary proteases.

In contrast the 4 amino acid substrates exhibit lower activity and higher specificity; as predicted from peptide library experiments. The ENaC-derived 4 amino acid substrates have demonstrated in vivo and vitro specificity. We have demonstrated nearly 100-fold higher activity of purified KLK1 for RKRR over the related peptide RKRK representing the two cleavage sites on gamma (Gondzik et al., Am J Physiol Cell Physiol. 2012 Nov 1;303(Adachi et al. 2006):C936–46. In vivo specificity is also in these peptides in our current and previous data from human and rat urine (Hu et al. 2009) indicating that that the single arginine to lysine substitution can cause large differences in activity, specificity and regulation; even though arginines and lysines are sometimes interchangeable serine protease substrates. In the end, it is possible that proteases which cleave RKRK and RKRR may also cleave other ENaC-unrelated proteins. However, given our data illustrating differences in activity with just a single amino acid substitution, it is likely that these substrates represent a reliable index of ENaC cleavage from the urinary space.

### Implication for disease

Many diseases such as metabolic syndrome and essential hypertension are accompanied or preceded by uncontrolled sodium retention, excess urinary sodium content and decreased pH. Furthermore, the urinary redox potential is also a variable (Grases et al. 2014). Clearly, the mere presence of these phenotypes implies a deranged ability to regulate sodium balance and especially absorption in the final portion of the nephron – the CD. Thus, a role for ENaC in these diseases can be deduced although a demonstration of ENaC dysfunction/mutation is limited. Given the large change in proteolytic activity we propose that salts, pH, ammonium, tricarboxylates, urea, metal ions, lipids, and redox potential could account for some of this derangement and inability to compensate.

Due to the degree of variability likely to be observed in urine from individuals with different degrees and subtypes of kidney disease, future studies that seek to examine the effects of kidney disease on protease activity will require larger groups of subjects and analysis of standardized urine.

### Implications for renal physiology

The strongest effects were observed with pH, Na^+^, and K^+^. The concentration of these ions is highly dynamic and varies physiologically and pathologically. Our data suggest a new pathway by which variability of these ions can modulate sodium absorption by effects on ENaC-acting protease and by extension ENaC activity. For example, high levels of urinary sodium could suppress ENaC activity partly through reduced proteolysis leading to enhanced Na^+^ excretion. Such regulation would enhance the known effects of Na^+^ to inhibit ENaC directly. Although the [Na] and [K] can vary between the medullary collecting duct and the cortical collecting duct, the collecting duct represents the largest site of regulated water absorption and by extension the largest site for variability in the concentration of molecules in its lumen.

We have recently established a functional link between the [Na] and ENaC cleavage demonstrating an inhibitory effect of high [Na] on ENaC activity brought about by reduced cleavage (Berman et al. 2015). A similar link has also been established in rats on high versus low Na diets (Palmer). Historically, the initial description of ENaC cleavage by Knepper and colleagues indicated an increase in a lower MW form of gamma ENaC (cleavage) under aldosterone conditions (Masilamani et al. 1999). In this case aldosterone which causes antinatriuresis increased cleaved gamma ENaC levels. Despite this observation, strong evidence linking aldosterone to changes in ENaC-acting proteases remains weak. This is maybe in part due to a complex effect of aldosterone on multiple proteases. Alternatively, the effect maybe mediated in part or whole by an effect of the decreased luminal sodium expected in the antinatriuresis. Additional future experiments correlating changes in activity in individual urine with aldosterone may provide the answer.

## Conclusions

The extent of changes to ENaC-acting protease are likely sufficient to cause marked change in in vivo channel activity. A multifold change in exogenous proteolytic activity can also cause a multifold activation of ENaC in cells, epithelia and isolated collecting ducts (Nesterov et al. 2008). Such changes would be more than sufficient to cause appreciable change in sodium balance. By analogy, the only known similar change in humans occurs in Liddle’s Syndrome. In these individuals, mutation of beta or gamma ENaC increase channel half-life at the membrane. In cells, epithelia, and transgenic mice these mutations cause 2–10-fold increase of ENaC activity (Firsov et al. 1996; Pradervand et al. 1999). This disease causes volume expansion and hypertension in humans despite the undoubted compensation by other renal transporters. We propose that these urinary components could provide a previously undiscovered mechanism for causing sustained changes of CD sodium absorption, and suggest that possible modification to the proteases themselves may also alter the way they respond to modulators. Irrespective, our data indicate that a simple static measurement will no longer be informative in correlating urinary protease activity with diseases of sodium absorption.
